# A comparison of subjective and objective measures of physical activity from the Newcastle 85+ study

**DOI:** 10.1093/ageing/afv062

**Published:** 2015-05-27

**Authors:** Paul Innerd, Michael Catt, Joanna Collerton, Karen Davies, Michael Trenell, Thomas B. L. Kirkwood, Carol Jagger

**Affiliations:** 1 Newcastle Institute for Ageing, Newcastle University, The Medical School, 4th Floor William Leech Building, Newcastle upon Tyne NE2 4HH, UK; 2 Institute of Health and Society, Newcastle University, Baddiley-Clark Building, Richardson Road, Newcastle upon Tyne, NE2 4AX, UK; 3 Institute of Cellular Medicine, Newcastle University, William Leech Building, Newcastle upon Tyne, NE2 4HH, UK; 4 Newcastle Institute for Ageing, Campus for Ageing and Vitality, Newcastle upon Tyne, NE4 5PL, UK

**Keywords:** physical activity, accelerometry, self-report, questionnaire, ‘aged 80 and over’, older people

## Abstract

**Background:** Little is known about physical activity (PA) in the very old, the fastest growing age group in the population. We aimed to examine the convergent validity of subjective and objective measures of PA in adults aged over 85 years.

**Methods:** A total of 484 participants aged 87–89 years recruited to the Newcastle 85+ study completed a purpose-designed physical activity questionnaire (PAQ), which categorised participants as mildly active, moderately active and very active. Out of them, 337 participants wore a triaxial, raw accelerometer on the right wrist over a 5–7-day period to obtain objective measures of rest/activity, PA intensity and PA type. Data from subjective and objective measurement methods were compared.

**Results:** Self-reported PA was significantly associated with objective measures of the daily sedentary time, low-intensity PA and activity type classified as sedentary, activities of daily living and walking. Objective measures of PA were significantly different when low, moderate and high self-reported PA categories were compared (all *P* < 0.001).

**Conclusion:** The Newcastle 85+ PAQ demonstrated convergent validity with objective measures of PA. Our findings suggest that this PAQ can be used in the very old to rank individuals according to their level of total PA.

## Introduction

There is compelling evidence that physical activity (PA) plays a major role in healthy ageing [1, 2]. In epidemiological research, PA is commonly assessed using physical activity questionnaires (PAQs) due to their practicality and low cost [3]. The design of the PAQ depends heavily on the population of interest [4]. In older populations, PAQs must be carefully designed to minimise recall bias [5] due to high rates of cognitive impairment [6] and to cover activities relevant for this age group. Since ageing is associated with functional decline [7] and a reduction in daily PA [8], the classification of total activity level may be more appropriate in the very old than more complex measurements such as energy expenditure [9].

Body-worn accelerometers provide objective measures of PA. Most accelerometers summarise the raw data into proprietary ‘counts’ [10] using methods kept confidential to the manufacturer [11]. However, raw accelerometers provide continuous acceleration data from which measures of PA can be derived using published algorithms [12, 13]. This increases methodological transparency and facilitates the comparison of data across studies.

PAQs are commonly compared with objective measures of PA from accelerometry to determine whether their outputs reflect similar parameters [14, 15]. However, none has done so for those aged 85 years and over, the fastest growing age group. The Newcastle 85+ study is the first to assess PA in the 85+ demographic using a PAQ and raw accelerometry. The aim of this study was to examine the convergent validity of a purpose-designed PAQ and raw accelerometry to assess PA in participants from the Newcastle 85+ study.

## Methods

The purpose of the main Newcastle 85+ study is to address key questions about the health trajectories of adults aged 85 years and over (see [16] for study protocol and [17] for baseline findings). Trained research nurses carried out data collection in the participant's place of residence at baseline (Phase 1: *n* = 849), at 18 months (Phase 2: *n* = 630) and then at 36 months (Phase 3: *n* = 484). Participants were invited to take part in the PA assessment involving PAQ and raw accelerometry as part of Phase 3.

### Subjective physical activity measures

A PAQ was designed using data from the Newcastle 85+ pilot study and then trialled in this age group prior to being implemented. The PAQ categorised participants into low (scores 0–1), moderate (scores 2–6) and high (scores 7–18) PA categories according to the frequency and intensity of PA carried out per week (Supplementary data Box S1, available in Age and Ageing online).

### Objective physical activity measures

Participants wore a triaxial, raw accelerometer (GENEA, Unilever, UK) continuously for 5–7 days on the right wrist. The technical specification of the GENEA has been described by van Hees *et al*. [18]. We derived the following measures of PA from the accelerometry data: mean acceleration (milli*g*) during the most active (M5) and least active (L5) 5-h period of each day and the difference between these periods (ΔM5L5); daily sedentary time (PA_SEDENTARY_) based on <1.5 METs (min/day) and low/moderate/high intensity PA (PA_LOW/MOD/HIGH_) based on ≥1.5 METs (min/day) [19]; and PA classified as sedentary behaviour (e.g. lying, sitting, standing), activities of daily living (ADL) (e.g. washing up, shelf stacking), walking and running [20].

## Statistical methods

The distribution of the data was checked using the Shapiro–Wilk test for normality (*P* < 0.05). As most of the data were non-normally distributed, non-parametric statistical tests were used. *P*-values were two sided and the level of significance set at 0.05. *T*-tests were used to test for significant differences between participants with and without full 5-day accelerometry data. Spearman's *ρ* correlation was used to test the strength of the association between variables derived from raw accelerometry and low, moderate and high self-reported PAQ categories. The Kruskal–Wallis non-parametric ANOVA test was used to test for significant differences between each of the measures derived from raw accelerometry for low, moderate and high self-reported PA. Missing values were excluded from analysis. All statistical analyses were carried out using SPSS version 21 (SPSS, Inc., Chicago, IL, USA).

## Results

All 484 participants in Phase 3 completed the PAQ. However, only 353 (73% of the 484) agreed to wear the accelerometer, and only 337 (70% of the 484) who had full 5–7-day accelerometry data were included in the analysis. Participants who agreed to wear the accelerometer were more likely to live in their own home (*P* < 0.001), had better cognitive function (*P* < 0.001), better self-rated health (*P* < 0.001) and lower disease count (*P* < 0.001). Participant demographics are shown in Supplementary data Table S1, available in Age and Ageing online.

**Figure 1.**
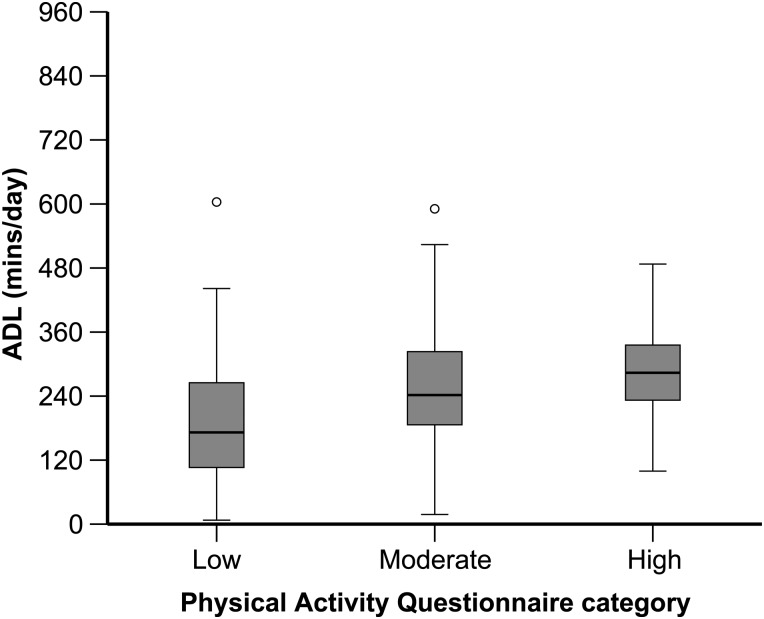
Average daily duration of ADLs derived from raw accelerometry for participants self-reporting low, moderate and high levels of PA. A time window of 960 min (16 h) was used for the *y*-axis.

Overall, participants self-reported mainly low (37%) or moderate (42%) PA levels whilst approximately half that amount reported high PA levels (21%). Raw accelerometry showed participants carried out approximately 3–5 h of ADLs per day (Figure 1).

When low, moderate and high PAQ categories were compared with objective PA measures using Spearman's rank correlation, modest significant correlations were found for M5 (0.10–0.32) and ΔM5L5 (0.09–0.33). L5 was not correlated with any PAQ category, most likely due to this period occurring during sleep. Modest significant correlations were found for PA_SEDENTARY_ (−0.10 to −0.33), PA_LOW/MOD/HIGH_ (0.10–0.34) and for PA type classified as sedentary (−0.21 to −0.32), ADL (0.11–0.29) and walking (0.11–0.52). No running was identified from the accelerometry data (Supplementary data Table S2, available in Age and Ageing online).

Participants reporting low, moderate and high PA levels had significantly different objective measures (Table 1), specifically, increases in M5 (*P* < 0.001), ΔM5L5 (*P* < 0.001), PA_LOW/MOD/HIGH_ (*P* < 0.001), PA classified as ADL (*P* < 0.001) and walking (*P* < 0.001), and decreases in L5 (*P* < 0.001), PA_SEDENTARY_ (*P* < 0.001) and PA classified as sedentary (*P* < 0.001).

**Table 1.** Comparison of PA measures from the questionnaire and accelerometry

	Low PA	Moderate PA	High PA	*P*-value^a^
	Mean ± SD	Mean ± SD	Mean ± SD	
Rest/active analysis (milli*g*)				
M5	21 ± 8	26 ± 7	31 ± 6	**<0.001**
L5	2 ± 1	2 ± 0.5	3 ± 1	0.20
ΔM5L5	19 ± 18	24 ± 7	27 ± 6	**<0.001**
Activity intensity classification (min/day)				
PA_SEDENTARY_	1335 ± 85	1283 ± 82	1241 ± 79	**<0.001**
PA_LOW/MOD/HIGH_	105 ± 85	157 ± 82	199 ± 79	**<0.001**
Activity type classification (min/day)				
Sedentary	1251 ± 112	1179 ± 96	1139 ± 93	**<0.001**
ADL	187 ± 112	255 ± 95	285 ± 89	**<0.001**
Walking	2 ± 4	6 ± 10	15 ± 17	**<0.001**
Running	0 ± 0	0 ± 0	0 ± 0	0.57

^a^The Kruskal–Wallis test derived *P*-value for significant differences between low, moderate and high self-reported PA.

Significant *P*-values are shown in bold.

## Discussion

This is the first study to compare subjective and objective measures of PA in adults aged over 85 years. The correlations between subjective and objective measures of PA (Spearman's *ρ* = 0.10–0.52), though modest, are comparable with those reported in a systematic review of studies involving adults up to the age of approximately 80 years [21] with the largest study to date (*n* = 2,721) reporting Spearman's *ρ* of 0.30 based on total self-reported PA [22]. A potential explanation for the modest correlations in our study is that the oldest old represent an extremely heterogeneous age demographic. Nevertheless, the measurement methods used in this study have several strengths.

Raw accelerometry allows several objective measures to be derived from the same acceleration signal. M5 and ΔM5L5 provide easy to interpret measures of activity that do not depend on existing physical capabilities of the population. This is pertinent to the 85+ age group as little is currently known about their functional capacity or metabolic demands [23]. Previous studies comparing PAQs and accelerometry often report difficulties in differentiating sedentary behaviour from low-intensity PA older populations [24, 25]. However, these studies typically use ‘traditional’ accelerometers that summarise the raw acceleration data into ‘counts’. The computational methods used to calculate counts depend on acceleration of the device exceeding an empirically derived threshold value over a given time window or ‘epoch’ [18]. The low-intensity short-duration PA of the elderly [26] that does not exceed this threshold may be poorly quantified. Therefore, the use of raw accelerometry may explain the differentiation of sedentary behaviour from low-intensity PA in this study. Raw accelerometry also allows the classification of PA by type [27]. With age, there is not only a reduction in PA intensity but also a change in activity type, where home-based activities and walking make up a larger proportion of physical activities [28]. Combined, these findings suggest that raw accelerometry provides robust objective measures of PA in the very old. However, a limitation of accelerometry in this age group appears to be a relatively low compliance (73%).

The PAQ used in our study was designed using data from the Newcastle 85+ pilot study and then trialled in this age group prior to being implemented in the main study. The design and delivery of the PAQ met criteria set out by Ainsworth and Casperson aimed at minimising measurement error in self-report measurement methods [29]. Firstly, the prevalence of cognitive decline in older people (34% in this cohort) meant that a concise, purpose-designed questionnaire was more appropriate for this study and could be answered by a proxy respondent. Secondly, questions featured examples of activities commonly carried out by older people [30]. Third, research nurses were trained in the delivery of the questionnaire, and finally, an objective measurement method was used for comparison. Benefits of the PAQ over accelerometry include its low cost and greater response rate, which is important when multiple assessments over time are required.

In conclusion, our results demonstrate convergent validity between subjective and objective measures of PA in the 85+ age group. Raw accelerometry provides objective measures of PA. However, compliance was low compared with our PAQ. The major strengths of our PAQ are that it is purpose designed for the 85+ demographic and more cost effective than accelerometry. These findings support the use of this PAQ in a very old population.

**Key points**

- Little is known about the assessment of PA in adults aged 85 years and over.
- This study compared subjective and objective PA measurement methods used in the Newcastle 85+ study.
- The results showed that our questionnaire demonstrated convergent validity with objective measures of PA.
- PAQs used in the 85+ age group should be concise and feature age-specific examples of activities.

## Supplementary data

Supplementary data mentioned in the text is available to subscribers in *Age and Ageing* online.

## Funding

The core Newcastle 85+ study was supported by a joint grant from the UK Medical Research Council and the Biotechnology and Biological Sciences Research Council (grant reference G0500997), the Dunhill Medical Trust (grant reference R124/0509) and NHS North of Tyne (Newcastle Primary Care Trust).

## Supplementary Material

Supplementary Data
