# Supplementary material for: A comparison of subjective and objective measures of physical activity from the Newcastle 85+ study
Source: Age Ageing. 2015 May 27;44(4):691–4. doi: 10.1093/ageing/afv062 (PMC4476851; doi:10.1093/ageing/afv062)
Supplement: Supplementary Data [file supp_44_4_691__index.html]

A comparison of subjective and objective measures of physical activity from the Newcastle 85+ study — Supplementary Data 

# A comparison of subjective and objective measures of physical activity from the Newcastle 85+ study

## Supplementary Data

Supplementary Data

- Supplementary Data - Docx file
