# Supplementary material for: A comparison of subjective and objective measures of physical activity from the Newcastle 85+ study
Source: Age Ageing. 2015 May 27;44(4):691–4. doi: 10.1093/ageing/afv062 (PMC4476851; doi:10.1093/ageing/afv062)
Supplement: Supplementary Data [file supp_afv062_afv062supp.docx]

**Supplementary online material**

**Box S1: Derivation of the subjective PA score**

The three PA items were:

A. How often do you take part in activities which are very energetic e.g. swimming, cycling, running or heavy gardening (digging with a spade, mowing the lawn (manual))?

B. How often do you take part in activities which are moderately energetic e.g. moderate gardening (raking, hoeing, mowing lawn (electric)), cleaning the car, heavy housework (cleaning windows, scrubbing floors), walking at a moderate pace, dancing, floor or stretching exercises?

C. How often do you take part in activities which are mildly energetic e.g. light gardening (weeding, pruning), bowls, light housework (vacuuming, washing clothes by hand, mopping floors, ironing, making beds), DIY?

Possible responses and scoring were:

- Hardly ever or never (score 0)
- Once, twice or three times a month (score 1)
- Once or twice a week (score 2)
- 3 or more times a week (score 3)

From the scores to the three questions the total PA score was calculated as 3*A+2*B+C.

Low physical activity (score 0-1)/ moderate physical activity (score 2-6)/ high physical activity (score 7-18).

| Table S1: **Participant characteristics** | |  |
| --- | --- | --- |
|  | | **Percent (n)** |
| Sex | Female | 63% (308) |
| Where living | Standard housing | 96% (466) |
|  | Institution | 4% (18) |
|  |  |  |
| Self-rated health | Excellent/very good | 35% (174) |
|  | Good | 33% (160) |
|  | Fair/poor | 19% (90) |
| Disability score | Fully independent (0) | 6% (31) |
|  | 1 - 6 | 44% (214) |
|  | 7 - 12 | 25% (123) |
|  | 13 - 17 | 12% (57) |
| Mini Mental State Examination score | No cog imp (26-30) | 64% (311) |
|  | Mild cog imp (22-25) | 17% (86) |
|  | Mod cog imp (18-21) | 8% (35) |
|  | Severe cog imp (0-17) | 9% (39) |
| Body Mass Index | <18.5 | 10% (49) |
|  | 18.5 - <25 | 43% (207) |
|  | 25 - <30 | 25% (120) |
|  | 30+ | 6% (28) |
| Presence of Disease | 1-2 | 10% (48) |
|  | 3-6 | 62% (352) |
|  | >7 | 10% (48) |

| Table S2: **Spearman’s rank correlation coefficients (*ρ*) for self-reported physical activity and measures derived from raw accelerometry** | | | |
| --- | --- | --- | --- |
| Accelerometer measures | Physical activity questionnaire category | | |
|  | Low activity | Moderate activity | High activity |
| M5 | 0.10* | 0.22** | 0.32*** |
| L5 | -0.3 | -0.7 | -0.8 |
| Δ M5L5 | 0.10* | 0.33** | 0.33** |
| PA_SEDENTARY_ | -0.10* | -0.38** | -0.33** |
| PA_LOW/MOD/HIGH_ | -0.10* | -0.38** | -0.34** |
| Sedentary | -0.21** | -0.30** | -0.32** |
| ADL | 0.11** | 0.28** | 0.29** |
| Walking | 0.15** | 0.48** | 0.52** |
| Running | - | - | - |
| *P<0.05 ** P<0.01 ***P<0.001 | | | |
